# Analysis of symptom clusters amongst adults with anorexia nervosa: Key severity indicators

**DOI:** 10.1016/j.psychres.2023.115272

**Published:** 2023-08

**Authors:** Zhuo Li, Jenni Leppanen, Jessica Webb, Philippa Croft, Sarah Byford, Kate Tchanturia

**Affiliations:** a King's College London, London, Department of Psychological Medicine, Institute of Psychiatry, Psychology, and Neuroscience, UK; b National Eating Disorders Service, South London and Maudsley NHS Foundation Trust, London, UK; c King's Health Economics, Health Service and Population Research Department, Institute of Psychiatry, Psychology & Neuroscience, King's College London, London, UK; d Psychological Set Research and Correction Center, Tbilisi State Medical University, Tbilisi, Georgia; e Department of Neuroimaging, Institute of Psychiatry, Psychology, and Neuroscience, King's College London, UK

**Keywords:** Anorexia nervosa, Eating disorder, Depression, Anxiety, Autism, Cluster analysis

## Abstract

This study used cluster analysis to explore clinically relevant subgroups of adult patients with anorexia nervosa (AN). Patients were clustered based on their body mass index (BMI), eating disorder symptomatology, anxiety and depression symptoms and autistic characteristics. The difference between clusters in work and social functioning, duration of illness, bingeing and purging behaviour, previous hospitalisations and number of comorbidities was also investigated. Two meaningful clusters emerged: a higher symptoms cluster with more severe eating pathology, anxiety, depression, and more autistic traits, and a second cluster with lower symptoms. BMI did not make major contributions to cluster formation. The higher symptoms cluster also reported lower self-efficacy to change, more previous hospitalisations, comorbid diagnoses, binge eating and purging behaviours and use of psychotropic medication. Our findings suggest that weight alone may not be a significant severity indicator amongst inpatients with AN, and targeted treatment of AN should consider a broader range of symptom severity indicators.

## Highlights

• Cluster analysis found groups of illness severity in adults with anorexia nervosa.
• The more severe group had more comorbidities, hospitalisations, and purging.
• Weight alone may not be a significant severity indicator.
• Treatment should consider a broader range of symptom severity indicators.

## 1 Introduction

Anorexia nervosa (AN) is a serious eating disorder (ED) with poor treatment outcome that can affect people of all ages, genders, and races (Schaumberg et al., 2017). Clinical decisions on the severity of AN are often guided by the patient's weight, as refusal to maintain healthy weight (given the patient's age and developmental stage) is a part of the diagnostic criteria for the illness. The 11th Edition of the International Classification of Diseases (ICD-11) (World Health Organization, 2019) has provided specific weight cut-offs and body mass index (BMI; kg/m^2^)-based severity indicators for AN, and the Diagnostic and Statistical Manual of Mental Disorders (5th ed.; DSM-5; American Psychiatric Association, 2013) also outlines weight criteria for AN. The Morgan-Russell outcome assessment schedule (Morgan and Hayward, 1988), which is often used in the clinical assessment of AN, defines patient outcomes based on body weight and menstrual function. Additionally, it has been reported that those with severe or extremely severe AN defined by BMI < 16.0 scored higher on measures of perfectionism and clinical impairment, suggesting that BMI is a crucial severity indicator in AN (Dakanalis et al., 2018). However, some studies have raised questions about the clinical validity of weight-based severity specifiers (Machado et al., 2017; Engelhardt et al., 2021; Toppino et al., 2022). One study reported that individuals who have lost a significant amount of weight but are not as emaciated as other patients with AN can still experience similar levels of life-threatening medical complications (Whitelaw et al., 2014). Other studies have found no significant evidence in favour of grouping AN patients into the BMI-based severity categories in terms of ED psychopathology or treatment outcomes (Machado et al., 2017; Toppino et al., 2022). Although low BMI remains a significant factor in AN, exploration of illness severity may benefit from including a wider range of psychological features.

AN has been reported to be a highly comorbid illness with some estimates suggesting that up to 97% of adult patients have at least one comorbid psychiatric diagnosis (Blinder et al., 2006; Marucci et al., 2018). The most common comorbid psychiatric diagnoses include depression and anxiety (Guinhut et al., 2021), which have been reported to be key factors in the development and maintenance of the AN (Lulé et al., 2014). Indeed, a recent network analysis by Monteleone and colleagues (2019) reported that depression and anxiety symptoms were central to the psychopathology of adolescent inpatients with AN. Another network analysis further documented that depression and anxiety symptoms, specifically feelings of worthlessness and avoidance of social eating, were not only strongly linked to core ED symptoms amongst adult AN patients, but also predicted recovery status at post-treatment follow-up (Elliott et al., 2020). Although most recent work has reported that severe depressive symptoms are associated with more severe AN pathology (Sternheim et al., 2015), worse treatment outcomes (Vall and Wade, 2015), and elevated risk of suicide (Kostro et al., 2014), there is some suggestion that moderate depression may have a positive association with weight gain and recovery in AN (Eskild-Jensen et al., 2020 for review). A large-scale study found that inpatients with AN who showed clinically significant improvements upon discharge were more likely to report moderate depression at admission when compared to deteriorated/unchanged patients (Schlegl et al., 2014). A similar effect of depression was found by Zeeck and colleagues, where inpatients with depression stayed longer in psychotherapy and may have a higher chance for clinically significant changes (Zeeck et al., 2005). These findings suggest that comorbid depression and anxiety are likely key factors contributing to illness severity in AN, but individual differences may also be present.

In addition to psychiatric comorbidities, recent evidence suggests that there is an over-representation of autism (Westwood and Tchanturia, 2017) and autistic features in AN (Kinnaird and Tchanturia, 2021). The estimated prevalence of autism or autistic characteristics in ED populations varies across studies from 22.9% to 36.2% (Wentz et al., 2005; Huke et al., 2013; Anckarsäter et al., 2012; Kinnaird et al., 2020; Vagni et al., 2016). Autistic patients often struggle with sensitivities to the sensory aspects of food, for example its smell, temperature, colour or texture (Leekam et al., 2007; Kinnaird et al., 2020) which may contribute to avoidance of certain food types in AN. Furthermore, both AN and autism are associated with high levels of alexithymia (Kinnaird and Tchanturia, 2021), interpersonal problems and social anxiety (Kerr-Gaffney et al., 2020), and neurocognitive aspects such as weak central coherence (Lang et al., 2014) and difficulties in set-shifting (Westwood et al., 2016). These findings warrant attention, as being autistic is often associated with greater use of intensive day-patient and inpatient treatment (Stewart et al., 2017; Nazar et al., 2018) and worse clinical outcomes in AN (Nielsen et al., 2015; Tchanturia et al., 2016).

Using exploratory, data-driven methods, such as cluster analysis, to explore patterns in a broad range of AN symptoms and severity indicators could help to shed light on the complexities in patients’ presentation and guide clinical decision making in treatment of AN. Cluster analysis explores patterns by grouping datapoints based on distance and thus can be used to identify subgroups in data without prior assumptions of the internal structure of the subgroups. Several previous studies have explored clustering of neuro- and social-cognitive measures, personality measures, and autistic features in adults with AN (Renwick et al., 2015; Rose et al., 2016; Bentz et al., 2020; Holliday et al., 2006). These studies have identified a variety of different clusters within the data used, but the clusters have not differed in ED symptoms, severity markers, or comorbidities, limiting the clinical implications of these findings. One study (Damiano et al., 2015) has examined clustering of behaviour and general psychopathology in adolescents with AN and identified two subgroups: one group that was underweight and scored lower on general and ED-specific psychopathology measures, and one group with higher general and ED psychopathology and higher BMI. This seems to be in contrast with previous finding that lower BMI predicts higher AN symptom severity (Löwe et al., 2001), but it is important to note that the sample size (*N* = 39) was small for cluster analysis. Interestingly, another study conducted a cluster analysis of a broad range of ED risk factors within a large community sample (Miles et al., 2022). Similar to the findings by Damiano and colleagues (2015), the authors identified low-, medium-, and high-risk groups, with the high-risk group reporting higher BMI and more depression and general ED symptoms than the medium- and low-risk groups. To our knowledge, no studies have yet used cluster analysis to explore subgroups in a broad range of illness severity indicators in a large sample of people with AN.

Based on the work outlined above, we explored patterns in a broad range of data, including information regarding BMI, ED psychopathology, common comorbid symptoms, and autistic features, which were collected from inpatients with AN upon admission. Given the exploratory nature of this approach, a number of research questions were posed in place of hypotheses:

(1) Can the analysis yield independent subgroups of patients that are not specific to the diagnostic criteria for AN, each with a different level of severity on the clustering variables?
(2) Are these subgroups different in other aspects of illness, such as duration of illness, bingeing and purging behaviour, number of hospital admissions, and number of comorbidities?

## 2 Methods

### 2.1 Participants

**Fig. 1:**
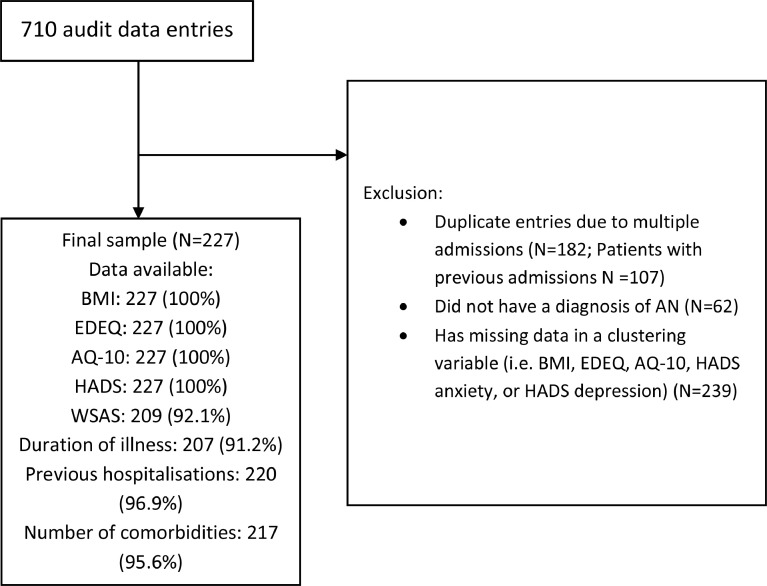
Data processing flowchart EDEQ: Eating Disorders Examination Questionnaire; AQ-10: Autism Spectrum Quotient, short version; HADS: Hospital Anxiety and Depression Scale; WSAS: Work and Social Adjustment Scale.

This study utilised clinical service audit data collected at the South London and Maudsley (SLaM) NHS inpatient ED service. As part of the clinical service audit, patients are asked to complete self-report questionnaires upon admission and their height and weight are recorded by a clinical team. Data from patients with a diagnosis of AN were included in the present study. Patients who did not complete the questionnaires that were used in the cluster analysis or for whom admission BMI was not available were excluded. If a given patient had multiple previous admissions, the admission with the most complete data was included to minimise missing data and other repeated admissions for the same person were excluded. A total of 107 patients had one or more readmissions, and 182 duplicate entries of admissions for these patients were therefore excluded. Out of the original 710 entries in the clinical service audit database, we ended up with a sample of 227 patients (Fig 1). The clinical service audit data collection and use were reviewed and approved by the Clinical Governance Committee Research and Development Office in South London and Maudsley (SLaM) NHS Trust in 2004.

### 2.2 Materials

#### 2.2.1 Measures used in cluster analysis

In addition to admission BMI recorded by members of staff, participants’ responses to self-report questionnaires assessing key aspects of AN and common comorbid symptoms were included in the cluster analysis. The self-report questionnaires included the Eating Disorder Examination Questionnaire (EDE-Q; Fairburn and Beglin, 1994), which is a self-report measure of behaviours and attitudes towards eating and body image and had excellent internal consistency (Cronbach's α = 0.96). To reduce dimensionality, the total score, which is given to summarise overall ED symptom severity, was included in the analysis. Participants also completed the Hospital Anxiety and Depression Scale (HADS; Zigmond and Snaith, 1983), which measures the severity of anxiety and depression symptoms in the week prior to admission with excellent internal consistency (Cronbach's α = 0.9). The short version of the Autism Spectrum Quotient (AQ-10; Allison et al., 2012) was used to screen for autistic features with acceptable internal consistency (Cronbach's α = 0.78). On all self-reported measures, higher scores indicate more severe symptoms.

#### 2.2.2 Measures not used in cluster analysis

The following measures were used to investigate differences between clusters that emerged from the current broad AN symptom profile. These included measures of general functioning, such as the Work and Social Adjustment Scale (WSAS; Mundt et al., 2002), which measures degree of everyday functional impairment with good internal consistency (Cronbach's α = 0.82). Items on the WSAS scale encompass different domains, including ability to work, home management, leisure activities, and ability to maintain close relationships. We also included data regarding participants’ age, their living situation (alone or with others), and the number of years they had faced unemployment due to their illness.

We also examined other indicators of illness severity and complexity including duration of AN, number of previous hospital admissions due to AN, number of comorbid diagnoses, and self-reported importance and ability to change ED behaviour on the Motivational Ruler (Miller and Rollnick, 2012). In addition to information regarding AN subtype, the open-ended questions in the EDE-Q regarding binge eating and purging behaviour were also included as additional markers of severity and complexity.

### 2.3 Data analysis

All data were analysed with R 4.1.0 (R Core Team, 2013). Admission BMI, EDE-Q total, AQ-10, HADS anxiety and depression scores were centred and scaled, and then entered into robust sparse k-means cluster analysis conducted using the *RSKC* package (Kondo et al., 2016; for the distance matrix plot, see Supplementary figure S1). We used robust sparse clustering to handle any potential outliers and reduce the impact of noise arising from any variables that didn't make strong contributions to cluster formation (Kondo et al., 2016). The silhouette method, as implemented in the *factoextra* package, was used to first determine the number of clusters and the final *RSKC* analysis was then conducted (Kassambara and Mundt, 2020) (Supplementary figure S2). The number of clusters was then confirmed using another package, *NbClust*, which utilises multiple indices (Charrad et al., 2014) (Supplementary figure S2).

The clusters were compared using the clustering measures (BMI, EDE-Q total, HADS anxiety, HADS depression, AQ-10) to evaluate which variables made strong contributions. The resulting clusters were then also compared using measures that were not included in the cluster analysis to determine if the clusters differed in other meaningful ways. These measures included the demographic and general functioning measures, as well as the illness severity and complexity measures. The cluster comparisons were conducted within the Bayesian framework using the *rstanarm* package, and probability of direction (PD) and the region of practical equivalence (ROPE) was estimated (Goodrich et al., 2022). Using Bayesian approach instead of frequentist statistics allowed for quantification of evidence strength, which increases the interpretability of observational clinical data. Additionally, Bayesian approach enabled us to evaluate whether the evidence was in favour of the alternative or the null hypotheses, which is not possible using frequentist approaches as even very large p-values cannot be taken as evidence in favour of the null hypothesis (Quintana and Williams, 2018). Differences in continuous variables, including WSAS score and illness duration, were analysed by conducting a Bayesian generalised linear regression, while count variables, such as number of previous hospital admissions and number of comorbid diagnoses, were subject to Bayesian generalised Poisson regression. Due to the heavily skewed nature of the data, binge eating and purging variables in the EDE-Q (‘How many such episodes have you had over the past four weeks’) were turned into binary variables (i.e. one or more episodes vs. no episodes). These and other binary variables were entered into Bayesian logistic regressions. Information regarding AN subtype was analysed by conducting a Bayesian analysis of contingency tables. *Weakly informative* priors were used in all analyses because information about the clusters was not known prior to analysis. Variable weights were calculated to inspect the importance of each variable in cluster formation, where the higher the variable weight, the more important the variable was in the partition of data. Bayes factors (BF) were calculated comparing the alternative (clusters are different) and null hypothesis (clusters are not different) using the *bayestestR* package (Makowski et al., 2019) to estimate the strength of the evidence. The Bayes factors were interpreted in accordance with Jeffreys’ (1961) proposed classification system (Table 1).

**Table 1:** Bayes factor interpretation table: classification of strength of evidence.

BF	Interpretation
> 100	Decisive evidence for the alternative hypothesis
30 – 100	Very strong evidence for the alternative hypothesis
10 – 30	Strong evidence for the alternative hypothesis
3 – 10	Moderate evidence for the alternative hypothesis
1 – 3	Anecdotal evidence for the alternative hypothesis
1	no evidence
1 – $\frac{1}{3}$	Anecdotal evidence for the null hypothesis
$\frac{1}{3}$ – $\frac{1}{10}$	Moderate evidence for the null hypothesis
$\frac{1}{10}$ – $\frac{1}{30}$	Strong evidence for the null hypothesis
$\frac{1}{30}$ – $\frac{1}{100}$	Very strong evidence for the null hypothesis
< $\frac{1}{100}$	Decisive evidence for the null hypothesis

BF = Bayes factor.

## 3 Results

### 3.1 Cluster characteristics

**Fig. 2:**
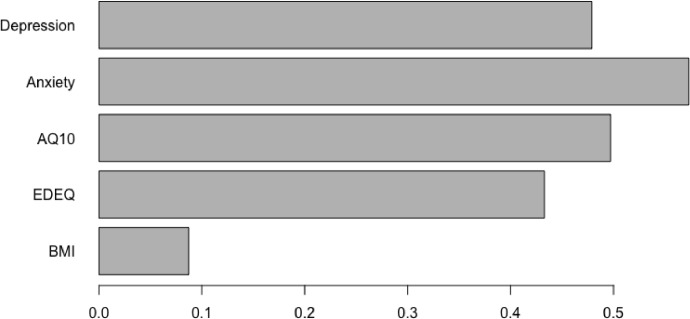
Relative contributing weights of clustering variables.

**Fig. 3:**
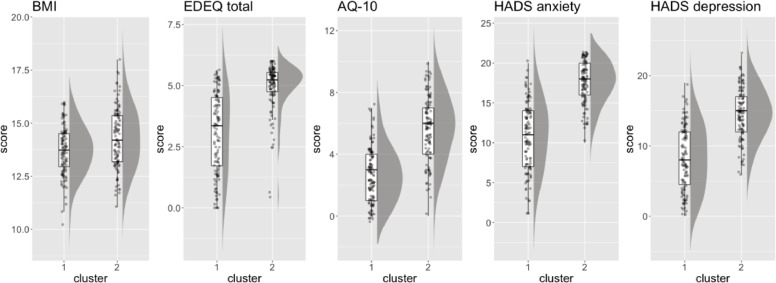
Differences between clusters in clustering variables BMI = body mass index; EDE-*Q* = eating disorder examination questionnaire; AQ-10 = autism spectrum quotient, short version; HADS = hospital anxiety and depressions scale.

The silhouette method indicated that there were two clusters present in the data (Supplementary figure S2). The clinical and demographic characteristics of the clusters are presented in Table 2 and Fig. 3. The clusters were of almost equal sizes with 115 (51%) patients forming cluster 1 and 112 (49%) forming cluster 2. There was decisive evidence to indicate that the patients in cluster 2 reported more ED symptoms, anxiety, depression, and autistic features than those in cluster 1. Cluster 2 is subsequently labelled “higher symptoms cluster”, and cluster 1 “lower symptoms cluster”. The clusters did also significantly differ on BMI, such that patients in cluster 2 had higher admission BMI than those in cluster 1. However, the evidence was only moderate suggesting that compared to the other measures admission BMI did not make substantial contributions to the cluster formation. This is further supported by observing the relative contributing weights of each cluster analysis variable (Fig. 2). As can be shown on Fig 2, BMI as a variable had the lowest contributing weight (0.09) in clustering, therefore the least important variable in the grouping of the data.

**Table 2:** Differences between clusters.

	Measure	Cluster 1 (*N* = 115)	Cluster 2 (*N* = 112)	Bayesian regression results			
				Median [95% CrI]	% in ROPE	PD	BF
Clustering variables	Admission BMI Mean (SD)	13.70 (1.13)	14.29 (1.41)	0.59 [0.26, 0.92]	0%	99.98%	4.35
	EDEQ total score Mean (SD)	3.07 (1.64)	4.95 (0.99)	1.88 [1.52, 2.23]	0%	>99.99%	4.39e+07
	AQ-10 score Mean (SD)	2.63 (1.71)	5.69 (2.07)	3.06 [2.56, 3.56]	0%	>99.99%	2.50e+13
	HADS anxiety score Mean (SD)	10.89 (4.21)	17.58 (2.61)	6.69 [5.77, 760]	0%	>99.99%	3.63e+14
	HADS depression score Mean (SD)	8.24 (4.54)	14.56 (3.59)	6.32 [5.24, 7.38]	0%	>99.99%	3.77e+09
General functioning and demographic variables	Age Mean (SD)	28.00 (12.01)	26.93 (9.01)	−1.08 [−3.84, 1.69]	45.26%	77.72%	1/46.07
	WSAS total Mean (SD)	24.89 (10.27)	26.88 (8.99)	1.99 [−0.65, 4.64]	21.37%	93.12%	1/16.79
	Years of unemployment due to AN Mean (SD)	12.21 (27.35)	10.46 (20.63)	−1.74 [−12.32, 8.80]	34.63%	62.89%	1/24.19
Illness severity and complexity variables	Duration of AN (years) Mean (SD)	8.98 (9.80)	10.20 (8.03)	1.23 [−1.25, 3.67]	36.67%	83.58%	1/33.08
	Medication use N (%)	54 (48.65%)	69 (64.49%)	0.65 [0.11, 1.21]	2.03%	99.05%	1/1.52
	Number of hospital admissions Mean (SD)	1.39 (2.39)	2.39 (3.86)	0.54 [0.34, 0.74]	0%	>99.99%	698.82
	Number of comorbidities Mean (SD)	0.66 (0.95)	1.25 (1.39)	0.64 [0.36, 0.93]	0%	>99.99%	113.18
	Binge eating N (%)	56 (48.70%)	82 (73.21%)	1.06 [0.51, 1.63]	0%	>99.99%	39.34
	Purging N (%)	35 (30.43%)	64 (57.14%)	1.12 [0.58, 1.67]	0%	>99.99%	69.92
	Motivational ruler: importance to change Mean (SD)	8.36 (2.34)	7.37 (2.47)	−0.98 [−1.64, −0.37]	0%	99.88%	1.45
	Motivational ruler: ability to change Mean (SD)	6.31 (2.99)	3.85 (2.69)	−2.46 [−3.19, −1.70]	0%	>99.99%	1.48e+04

BMI = body mass index; EDEQ = eating disorder examination questionnaire; AQ-10 = autism spectrum quotient, short version; HADS = hospital anxiety and depressions scale; WSAS = work and social adjustment scale; SD = standard deviation; CrI = credible interval; ROPE = region of practical equivalence; PD = probability of direction; BF = Bayes factor.

### 3.2 General functioning and demographic characteristics

The clusters did not differ significantly in WSAS scores, the number of years the patients had been unemployed due to their illness, or in age. In fact, there was strong to very strong evidence in favour of the null hypothesis. The clusters also did not differ in the distribution of AN diagnostic subtypes (Table 3), with strong evidence in favour of the null hypothesis (BF = 1/22.26).

**Table 3:** Distribution of AN diagnostic subtypes between the clusters.

	Cluster 1 (*N* = 115)	Cluster 2 (*N* = 112)
AN Restrictive, N (%)	84 (73.04%)	80 (71.43%)
AN Binge-purge, N (%)	26 (22.61%)	29 (25.89%)
AN Atypical, N (%)	5 (4.35%)	3 (2.68%)

### 3.3 Illness severity and complexity

There was no significant difference between the clusters in duration of illness but there was decisive evidence that patients in cluster 2 had experienced significantly more previous hospitalisations due to AN than those in cluster 1. Additionally, patients in cluster 2 also reported lower self-efficacy in their ability to change, and more complex presentation including more comorbid diagnoses and a greater tendency to binge and purge than those in cluster 1 with very strong to decisive evidence. There was also a significant difference between the clusters in the self-reported use of psychotropic medication on admission, such that a higher proportion of patients in cluster 2 were taking medication on admission. However, there was only anecdotal evidence for this difference (BF = 1/1.52), suggesting no firm conclusions about medication use can be drawn based on the present data. Similarly, there was a significant difference between the clusters in motivation to change, but the evidence for this difference was only anecdotal (BF = 1.45), suggesting no firm conclusion about this variable can be drawn based on the observed data.

## 4 Discussion

This study aimed to derive clinically distinct subgroups of adult patients with AN through a data-driven clustering approach. Patients were clustered based on their BMI, self-report eating pathology and general psychopathology. Amongst the two resulting clusters of similar sizes, cluster 2 ("higher symptoms cluster”) reported higher scores on eating pathology, anxiety, depression, and autism screening than cluster 1 ("lower symptoms cluster”). The higher symptoms cluster also reported lower self-efficacy to change, more previous hospitalisations, comorbid diagnoses, binge eating and purging behaviours and use of psychotropic medication than the lower symptoms cluster. It should be highlighted that although the higher symptoms cluster also had slightly higher admission BMI, our inpatient sample overall had very low weight and BMI did not make major contributions to cluster formation.

### 4.1 The higher symptoms cluster

We found that the higher symptoms cluster scored high on all clustering variables, as well as on several illness severity and complexity indicators, such as binge and purge behaviours and number of comorbidities. Furthermore, the elevated autistic characteristics in this cluster are consistent with previous research suggesting an over-representation of autism in EDs (Westwood and Tchanturia, 2017). In particular, our results are in line with the literature highlighting emotional difficulties in autistic women with AN (Brede et al., 2020), which often relate to exacerbated anxiety and depression (Tchanturia et al., 2019). The maladaptive presentation and increased hospitalisations in this cluster also confirm previous research where individuals with co-occurring autism and EDs often present with worse treatment outcomes (Nielsen et al., 2015) and increased service use (Nazar et al., 2018). Similarly, a previous study clustering individuals with AN based on neuropsychological features (i.e. executive function, central coherence, and theory of mind) also identified an autism-like subset with difficulties in executive function and central coherence (Renwick et al., 2015). This link between autistic characteristics and illness severity warrants clinical attention, as autistic individuals’ sensory and cognitive profile may make it more difficult for them to benefit from standard ED treatment designed for neurotypical patients, which calls for treatment adaptations and innovations for autistic patients or those with autistic traits.

Previous empirical work has also highlighted that purging behaviour is associated with negative outcomes, comorbidities, and life-threatening physical complications such as electrolyte disturbances (Keel et al., 2004; Solmi et al., 2015; Royal College of Psychiatrists, 2022). Indeed, the higher symptoms cluster in the current study presented with more binge and purge behaviours in combination with more negative affect. Notably, patients in this cluster also had more previous hospital admissions, despite having similar duration of illness as the lower symptoms cluster. This finding is in direct contrast with previous work which has argued that duration of illness is a key severity indicator (Maguire et al., 2012). On the other hand, this is in line with previous findings linking binge-purge behaviours and low affect with treatment resistance in severe AN (Smith and Woodside, 2021; Di Lodovico et al., 2021). Interestingly, despite one cluster reporting more binge-purge behaviours than the other, the two clusters did not differ in the proportion of individuals with AN binge-purge diagnosis. It is possible that for some patients, their frequency of binge and purge behaviours simply did not meet the clinical cut-off for receiving a diagnosis. Our results show that regardless of the level of engagement, binge-purge behaviour is linked to more complex and severe presentation and therefore needs clinical attention. Another possible explanation is that individuals reported binge-purge behaviours on the questionnaire but denied these behaviours when questioned in a diagnostic interview and therefore did not receive a binge-purge subtype diagnosis. This is in line with previous finding that approximately 40% of participants who reported purging behaviour on self-report measures subsequently denied this in a face-to-face interview (Mond et al., 2007), possibly due to a greater shame in disclosing purging behaviours when faced directly with an interviewer. This inconsistency between self-report and clinical assessment should be addressed with caution to prevent problematic behaviours like purging from being missed by the clinical team.

We also found that the higher symptoms cluster had significantly higher, not lower, admission BMI (Mean=14.29, SD=1.41) than those in the lower symptoms cluster (Mean=13.70, SD=1.13). This is in line with previous clustering studies using larger (Miles et al., 2022) and smaller (Damiano et al., 2015) samples of patients with ED which identified high symptom groups reporting higher BMI. However, it is important to consider that this statistically significant difference may not be clinically meaningful. Indeed, the associated Bayes factor (BF=4.35) and cluster weight (0.09) of the BMI variable suggest that admission BMI did not make substantial contributions to the cluster formation. Together, the findings suggest that weight alone may not be a significant symptom severity indicator amongst inpatients with AN, most of whom have very low BMI. This is consistent with previous work showing that improvement in psychopathology in AN does not correlate with BMI improvement (Mattar et al., 2012), suggesting for a better indicator for illness severity such as purging behaviour and comorbid symptoms, rather than BMI alone. Furthermore, this finding brings attention to individuals who have lost a significant amount of weight but may still be at a higher weight than other patients. These patients are commonly diagnosed with atypical AN or Eating Disorder Not Otherwise Specified (EDNOS) (Moskowitz and Weiselberg, 2017). Despite not being as emaciated as patients who are more underweight, these patients can experience a similar profile of life-threatening complications (Whitelaw et al., 2014) and deserve just as much clinical attention.

### 4.2 The lower symptoms cluster

With lower scores on the self-report measures, individuals in the lower symptoms cluster appear to have better functioning and fewer difficulties with eating and general psychopathology. This is in line with previous findings that individuals reporting less fear of weight gain and ED symptoms also appeared to have less severe psychopathology (Ramacciotti et al., 2002). However, it is also possible that patients in this cluster are simply more used to suppressing their emotions, a problem that is most pronounced in the acute phase of the illness (Oldershaw et al., 2015), thereby leading to lower self-reported symptoms. It has been suggested that starvation may at least partly serve as a strategy to regulate unwanted emotions and feelings (Haynos and Fruzzetti, 2011). Starvation numbs both physiological and emotional responses thus provides escape or a safe place, but it also potentially makes it more difficult to describe or identify own internal states (Malova and Dunleavy, 2022; Lavis, 2018; Oldershaw et al., 2015; Rowsell et al., 2016). Therefore, the lower symptoms reported in this cluster might in fact be a warning sign of emotional avoidance. On the other hand, it is also possible that the lower scores on self-report measures were due to denial of symptoms, particularly on the EDE-Q where some patients reported next to no ED symptoms. Deliberate denial and distortion of symptoms are common in AN due to body image disturbance and resistance to change (Vitousek et al., 1991). This also reflects reduced insight and low self-awareness in this cluster, which may dangerously lead to a more difficult recovery path (Errichiello et al., 2016). Longitudinal research would be of interest to shed light on the underlying mechanisms and outcomes for under-reported symptoms amongst inpatients with AN.

### 4.3 Limitations

One limitation of this study is the use of self-report questionnaires alone. As previously discussed, it is possible for individuals to deny or minimise symptoms in self-report measures such as the EDE-Q. Moreover, the accuracy of self-report autism screening tools such as the AQ-10 in the ED population has been controversial. The internal consistency for AQ-10 in this study (Cronbach's α = 0.78) was acceptable but not excellent. Future studies should consider combining assessment interviews with self-report measures of ED symptoms, as well as more rigorous measures of autistic characteristics. Another limitation of the study was the number of patient entries excluded due to missing data; although patients who were included in the end had complete data in all of the clustering variables, there was missing data in other clinical and demographic variables not used in clustering. This was inevitable, given that the study used observational clinical audit data. However, this does not affect the robustness of the clustering, and Bayesian inference was used for its enhanced interpretability in observational data. Lastly, due to demographic constraint of the inpatient setting, the current study only examines inpatients mostly with severe AN. Any conclusions on the significance of weight, purging, autistic characteristics, or negative affect would need to be validated within a larger cohort of people with a broader range of ED severity and subtypes.

## 5 Conclusions

This study suggests that distinct groups of illness severity exist in adults with AN. More complex and severe presentation in AN is associated with more comorbidities (including autism, anxiety and depression), previous hospitalisations, binge eating and purging behaviours and use of psychotropic medication. BMI did not make major contributions to the clustering, suggesting that weight alone may not be a significant severity indicator. Our findings warrant future studies that investigate aetiological categorisation including other ED populations (e.g., bulimia nervosa) and promote the use of a broader range of validators to guide treatment tailoring in ED.

## Funding

This research was funded in part by the Wellcome Trust [213,578/Z/18/Z]. The research was further supported by 10.13039/501100007155MRC-MRF Fund [MR/R004595/1]. For the purpose of open access, the author has applied a CC BY public copyright licence to any Author Accepted Manuscript version arising from this submission.

## CRediT authorship contribution statement

**Zhuo Li:** Data curation, Writing – original draft, Writing – review & editing. **Jenni Leppanen:** Formal analysis, Writing – original draft, Writing – review & editing. **Jessica Webb:** Data curation, Writing – review & editing. **Philippa Croft:** Data curation, Writing – review & editing. **Sarah Byford:** Supervision, Writing – review & editing. **Kate Tchanturia:** Supervision, Writing – review & editing.

## Declaration of Competing Interest

The authors have no conflicts of interest to declare that are relevant to the content of this article.

## Appendix Supplementary materials

Image, application 1
