# Supplementary material for: Analysis of symptom clusters amongst adults with anorexia nervosa: Key severity indicators
Source: Psychiatry Res. 2023 Aug;326:115272. doi: 10.1016/j.psychres.2023.115272 (PMC10790244; doi:10.1016/j.psychres.2023.115272)

S1. Distance matrix plot


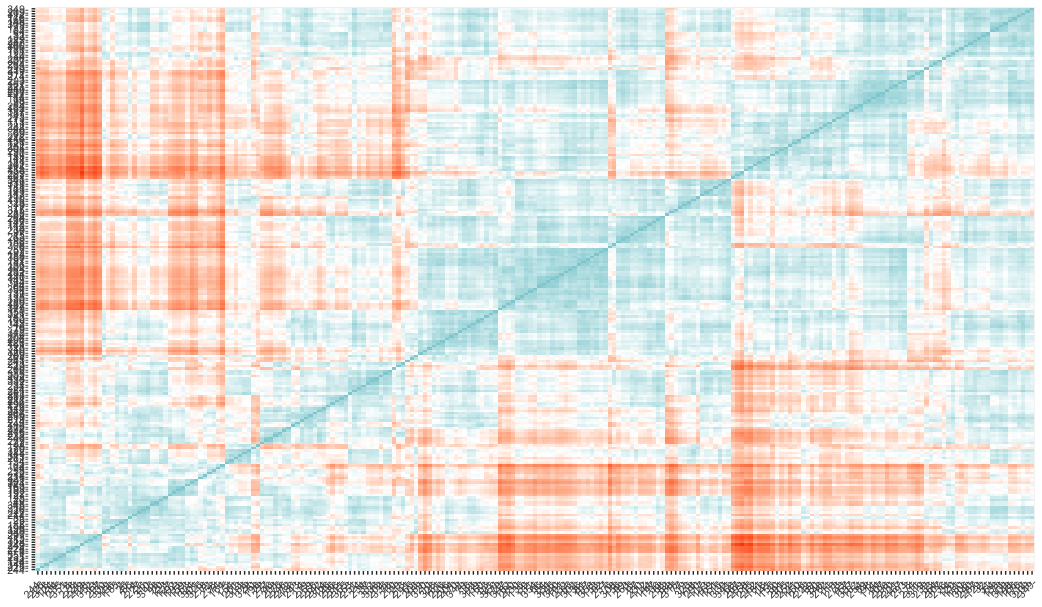


S2. Silhouette plot for the number of clusters and confirmation using NbClust


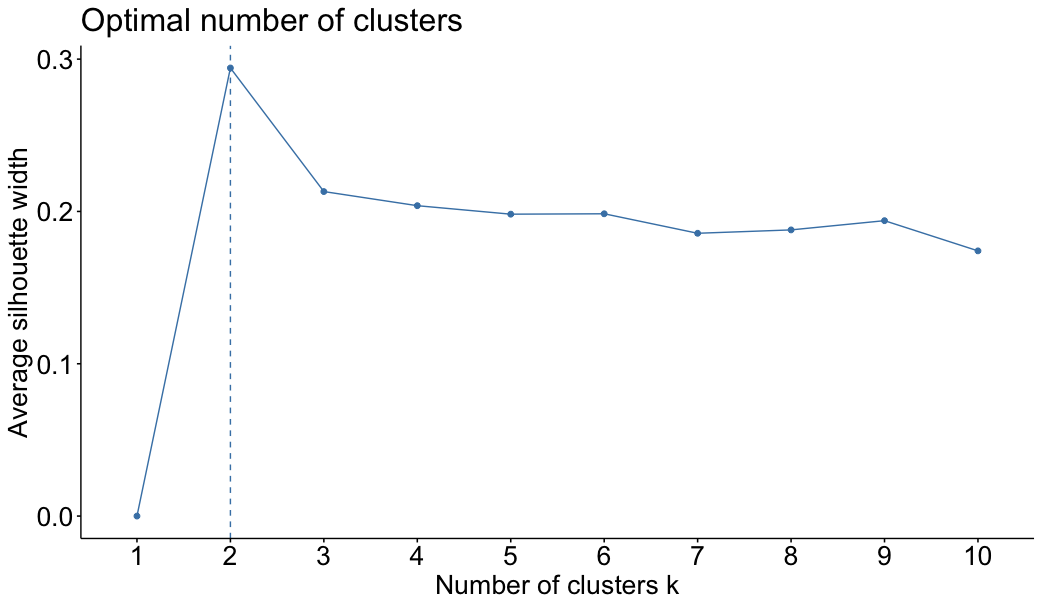


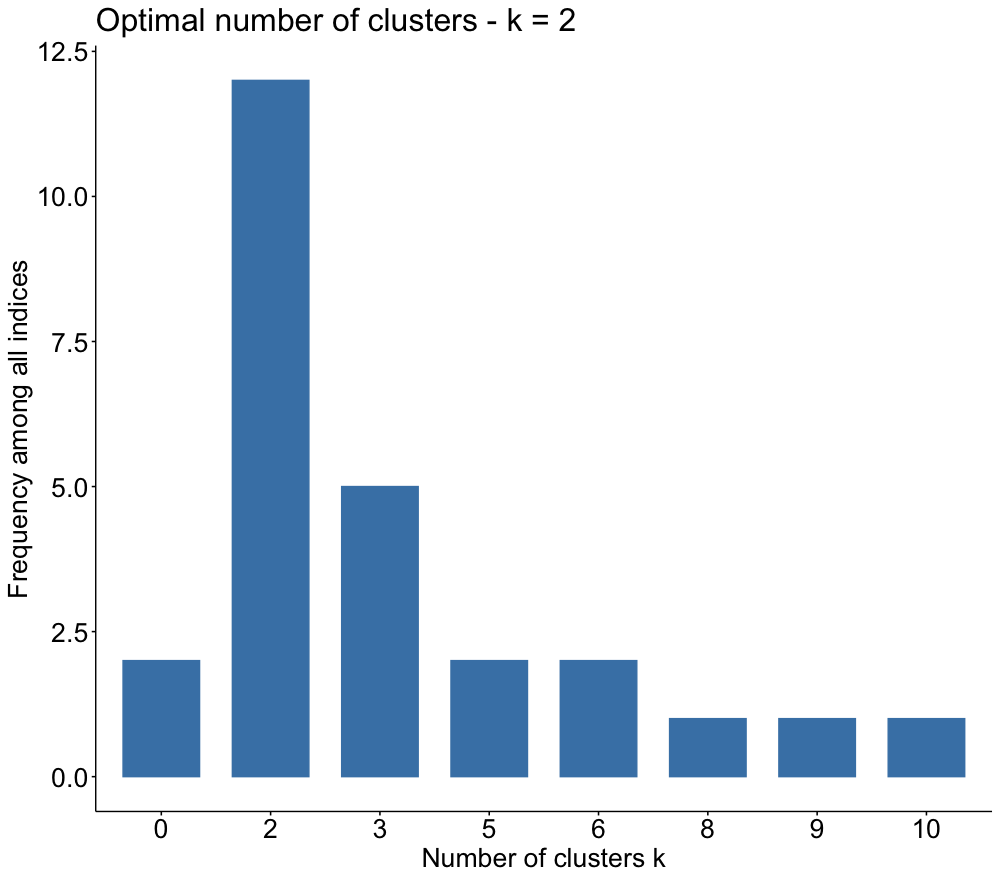

Supplement: Supplementary file 1 [file mmc1.docx]
